# Toward Individualized Management: A Commentary on Perioperative Systemic Therapy Guidelines in Breast Cancer Surgery and Reconstruction. Comment on Galuia et al. Perioperative Drug Management of Systemic Therapies in Breast Cancer: A Literature Review and Treatment Recommendations. *Curr. Oncol.* 2025, *32*, 154

**DOI:** 10.3390/curroncol33050294

**Published:** 2026-05-19

**Authors:** Emily E. Zona, Jacqueline S. Israel

**Affiliations:** Division of Plastic and Reconstructive Surgery, University of Wisconsin School of Medicine and Public Health, Madison, WI 53792, USA; ezona@wisc.edu

## Abstract

The perioperative management of systemic breast cancer therapies is an increasingly important aspect of planning for breast cancer surgery and reconstruction. This commentary compares two recent sets of recommendations and explains why treatment decisions often need to be tailored to the individual patient. By highlighting differences in methodology and clinical considerations, we emphasize the importance of individualized perioperative planning and close collaboration between surgeons and oncologists. Continued research will help refine these strategies and improve care for patients undergoing breast reconstruction.

## 1. Introduction

We read with great interest the article by Galuia et al. in *Current Oncology*, which offers a thoughtful and comprehensive synthesis of perioperative considerations for systemic therapies in breast cancer patients undergoing oncologic extirpative surgery, reconstruction, or both [1]. We appreciate the authors’ synthesis of pharmacokinetic principles, preclinical data, and other peer-reviewed literature into practical perioperative guidelines, particularly in an area where guidance has historically been fragmented and inconsistent.

Our group recently published a related article, “Anticancer Agents and Their Impact on Breast Reconstruction: A Guide for Plastic Surgeons Based on Systematic Review and Expert Consensus”, in *Plastic and Reconstructive Surgery* [2]. Our aim was similar: to clarify perioperative management strategies for commonly prescribed systemic therapies in patients with breast cancer undergoing reconstructive procedures through systematic review and multidisciplinary expert consensus. While we arrived at some different recommendations than Galuia et al., we share their overarching conclusion: there is no universally accepted standard for the perioperative management of systemic breast cancer therapies, and, as such, clinical decision-making remains variable, nuanced, and often institution-specific, shaped by institutional culture and surgeon–oncologist collaboration. Like in other clinical scenarios, we believe this is where the art of medical decision-making and available evidence intersect.

## 2. Discussion

Where our two publications diverge is in application. Galuia et al. propose perioperative drug holds primarily based on pharmacokinetics, specifically drug elimination half-lives, in the absence of preclinical or clinical wound-healing data. This is a great point, and we feel that recommendations can be generated from both the pharmacokinetic data as well as from collective expert consensus gleaned from clinical experience. As an example, neutropenia and thrombocytopenia may persist well beyond the pharmacologic clearance of the drug. These effects may significantly impact surgical planning and postoperative recovery, particularly in major autologous breast reconstruction procedures where the patient’s immune system is being asked to heal surgical wounds at multiple sites [3,4]. For example, a patient who has technically cleared a CDK4/6 inhibitor may still have an absolute neutrophil count (ANC) less than 1000, placing them at elevated risk of infection or impaired wound healing. We recommend routine preoperative complete blood count (CBC) testing with differential for select agents such as abemaciclib [5], ado-trastuzumab emtansine, capecitabine, and Olaparib, and suggest delaying surgery if neutropenia or thrombocytopenia is present [2,6,7]. These recommendations reflect expert multidisciplinary consensus drawn from the available data demonstrating hematologic risks with these agents, in the absence of reconstruction-specific prospective evidence.

We also find it important to stratify recommendations by surgical complexity. The terms “breast surgery” and “breast reconstruction” encompass a wide spectrum of procedures with different risk profiles. For example, the implications of perioperative systemic therapy use differ substantially between a DIEP flap requiring abdominal wall closure and fragile microsurgical anastomoses versus a relatively minor revision surgery such as fat grafting or nipple reconstruction. For minor procedures such as these, we do not recommend holding any anticancer medications unless thrombocytopenia is a concern. To better contextualize risk, our guidelines distinguish between major and minor procedures, applying drug-hold recommendations to major breast reconstruction surgeries like autologous reconstruction, and allowing greater flexibility for lower-risk revisions or secondary procedures. Whether reconstruction is performed immediately at the time of mastectomy or in a delayed fashion represents an additional layer of complexity that may further influence perioperative drug-hold decisions. We believe this distinction warrants dedicated prospective investigation and was not addressed in depth by either our group or Galuia et al.

Galuia et al. also differ from us in the duration and breadth of drug holds they suggest. This difference likely reflects how we interpreted the available data regarding these medications and underscores that more data is needed. We found that our recommendations for hold times are typically shorter (1–2 weeks) and are individualized based on patient factors and lab results. We believe that this tailored approach allows for continuity of essential cancer therapies when safe, without compromising surgical outcomes.

Ultimately, both articles highlight the tension between rapidly evolving systemic cancer therapies and advancements in breast reconstruction practices. In the absence of robust prospective trials, clinical protocols must rely on a combination of pharmacologic reasoning, retrospective data, and, critically, interdisciplinary expert discussion. We are encouraged to see other groups tackling this issue. While our methodologies and interpretations may differ slightly, the message is clear: there is no single “correct” way to perioperatively manage these anticancer agents, and diverse approaches should be welcomed as part of a broader dialogue aimed at optimizing outcomes. We hope this exchange catalyzes further cross-specialty discussion and prospective research. Until more robust data are available, interdisciplinary team-based decision-making remains our best strategy.

## Abbreviations

The following abbreviations are used in this manuscript:

ANC	Absolute neutrophil count
CBC	Complete blood count
DIEP	Deep inferior epigastric perforator
